# Effects of a pressure-ulcer audit and feedback regional programme at 1 and 2 years in nursing homes: A prospective longitudinal study

**DOI:** 10.1371/journal.pone.0233471

**Published:** 2020-05-29

**Authors:** Lorenzo Righi, Aimad Ourahmoune, Nadine Béné, Anne-Claire Rae, Delphine S. Courvoisier, Pierre Chopard

**Affiliations:** 1 Quality of Care Unit, University Hospitals of Geneva, Geneva, Switzerland; 2 Department of General Internal Medicine, Rehabilitation and Geriatrics, University of Geneva, Geneva, Switzerland; 3 Quality of Care and Clinical Networks, Tuscany Region, Italy; 4 Geneva Nursing Homes Association, Geneva, Switzerland; 5 Health Care Research and Quality, University Hospitals of Geneva, Geneva, Switzerland; University Magna Graecia of Catanzaro, ITALY

## Abstract

**Introduction:**

Pressure ulcer is a frequent complication in patients hospitalized in nursing homes and has a serious impact on quality of life and overall health. Moreover, ulcer treatment is highly expensive. Several studies have shown that pressure ulcer prevention is cost-effective. Audit and feedback programmes can help improve professional practices in pressure ulcer prevention and thus reduce their occurrence. The aim of this study was to analyze, with a prospective longitudinal study, the effectiveness of an audit and feedback programme at 1- and 2-year follow-up for reducing pressure ulcer prevalence and enhancing adherence to preventive practices in nursing homes.

**Methods:**

Pressure ulcer point prevalence and preventive practices were measured in 2015, 2016 and 2017 in nursing homes of the Canton of Geneva (Switzerland). Oral and written feedback was provided 2 months after every survey to nursing home reference nurses.

**Results:**

A total of 27 nursing homes participated in the programme in 2015 and 2016 (4607 patients) and 15 continued in 2017 (1357 patients). Patients were mostly females, with mean age > 86 years and median length of stay about 2 years. The programme significantly improved two preventive measures: patient repositioning and anti-decubitus bed or mattress. It also reduced acquired pressure ulcers prevalence in nursing homes that participated during all 3 years (from 4.5% in 2015 to 2.9% in 2017, p 0.035), especially in those with more patients with pressure ulcers.

**Conclusion:**

Audit and feedback is relatively easy to implement at the regional level in nursing homes and can enhance adherence to preventive measures and reduce pressure ulcers prevalence in the homes.

## Introduction

Pressure ulcer (PU), or pressure injury, is a significant complication in many patient populations and all healthcare settings. It is defined as a localized injury to the skin and/or underlying tissue usually over a bony prominence as a result of pressure or pressure combined with shear [1]. PU causes substantial physical, psychological, functional and social burden to patients, especially older patients [2–4]. PU treatment is expensive and directly proportional to severity: £1,214 for category (stage) 1 (low severity) PU to £14,108 for category 4 (highest severity) PU [5].

PU onset is often avoidable and can be prevented. Using support surfaces, repositioning the patient, optimizing nutritional status, and moisturizing pressure-bearing surfaces are generally considered effective preventive strategies [6]. Prevention is cost-effective, especially for more severe cases. According to a recent meta-analysis [7], PU prevention in long-term care institutions costs from 2.65 and 19.69 €/day, whereas the cost of PU treatment per patient per day ranges from 2.16 € for a category 1 PU to 170.43 € for a category 4 PU.

Optimal PU rate has been defined as less than 2%, but it may vary depending on patient case mix, severity of illness, and other contextual factors [8]. In nursing homes, PU prevalence ranges from 2.2% to 23.9% [8]. Among high-risk chronic-care residents, the prevalence is higher, from 3% to 33% [9]. Similar results were also found in the Canton of Geneva (Switzerland) [10]. Such a wide variability indicates that PU prevention and care are suboptimal. In a seminal study, Wennberg and Gittelsohn demonstrated a huge unwarranted variation in health services utilisation [11]. Adherence to recommended processes for basic care is still a problem in the third millennium: the proportion of recommended care provided is just under 55% in the United States [12] and 57% in Australia [13]. Evidence for variation and underuse of effective clinical practices exists in all countries and at all stages along the care continuum [14].

Audit and feedback is a widely used quality improvement process. The underlying idea is that the quality and safety of healthcare might be improved if healthcare professionals are given information about their clinical performance, thereby allowing them to assess and adjust their performance [15]. The approach generally leads to small but potentially important improvements in professional practice [16] and was mentioned as a key element in 20 of 26 (80%) PU preventive initiatives in acute and long-term care settings [17].

The objective of the present study was to analyze the effectiveness of an audit and feedback programme for enhancing adherence to preventive practices and reducing facility-acquired PU prevalence in a multicenter study of nursing homes followed for 2 years. A secondary objective was to provide a first estimate of the costs of such a programme in terms of time and effort.

## Materials and methods

### Study design

The present study was based on a prospective longitudinal design with cross-sectional assessments in 2015, 2016, and 2017.

### Procedure

One reference nurse per nursing home received a 2-hr training in data reporting (every item was explained; Braden Scale and subscales, preventive measures, and PU categories were reviewed with real-life examples and visual material).

PUs were assessed in November 2015, 2016 and 2017 by the reference nurse, who collected information on socio-demographic and clinical characteristics, Braden score, prevention practices, and presence, origin and severity of PU for each patient.

### Measures

Skin and Braden Scale assessment was completed on the day of the investigation. All other variables were collected by using patient records. The Braden Scale is a tool for predicting PU risk by examining six criteria: sensory perception, moisture, activity, mobility, nutrition and friction/shear. Patients were considered at high risk with Braden score <15 and at low risk with Braden score ≥ 15 [18].

To determine which PU was “facility-acquired”, patient records were reviewed. If several PUs were found, only the most severe was considered. Unstageable pressure injuries or suspected deep-tissue injuries were included as category (stage) 4 PU.

For each patient, the number of preventive measures according to Braden Scale and subscales was computed. Four preventive measures were considered: oral nutrition supplement administration, patient repositioning, the presence of an anti-decubitus bed or mattress, and moisturizing cream administration. Oral nutrition supplement was considered appropriate with a Braden nutrition subscore 1 or 2. Repositioning-recommended frequency was based on the Braden risk score, regardless of the anti-decubitus bed or mattress: every 2 to 3 hr for very high and high-risk patients and every 4 hr for moderate-risk patients; repositioning was not considered necessary for the other patients. An anti-decubitus bed or mattress and moisturizing cream were considered appropriate for all patients at risk (score ≤ 18). The “moisturizing cream” item was not present in the 2015 survey.

### Audit and feedback

Two months after the annual survey, during a same day meeting, the programme coordinator gave both oral and written feedback to nursing-home reference nurses. Two types of written reports were provided. The first report presented the overall prevalence of preventive measures, including acquired-PU prevalence per Braden risk level and treatment strategies per PU severity. This first report was given to all nursing homes and was also discussed orally. The second report was specific for each nursing home and was given only to the nursing home concerned. It showed the same overall indicators and contrasted them with the indicators for the specific nursing home.

In the following days, the two reports were sent by e-mail to nursing home directors and were discussed with nursing home staff members. Telephone availability to explain and complete the results of each nursing home was guaranteed.

### Statistical analysis

Descriptive data are presented with frequency (%) for categorical variables and median and interquartile range (IQR) for continuous variables. Exact two-sided binomial test with the central method was used to calculate 95% confidence intervals (CIs) for preventive measures and acquired PU. To estimate the effectiveness of the program at 1 and 2 years in reducing PU prevalence and increasing adherence to preventive practices, we used the Mantel–Haenszel test adjusted for nursing home and Braden risk level. The Mantel–Haenszel test was also used to evaluate the effect of nursing home size and length of stay on the same outcomes: the test was performed considering the number of acquired PUs, Braden Scale score and nursing home size (classified as large, >80 beds; medium, 80–60 beds; and small, <60 beds) or length of stay (classified in quartiles: first quartile, <1 year; second quartile, 1–2 years; third quartile, 2–4 years; fourth quartile, 4–31 years). Staff training, occupancy rate, and profit status were similar in all nursing homes and thus could not be adjusted for. Nursing homes reported that clinical staff did not change qualitatively or quantitatively during the study period.

According to the policy activities that constitute research in the Geneva state, this work met criteria for operational improvement activities exempt from ethics review.

All analyses were performed with R v3.4.2 (https://www.r-project.org).

### Costs

The time required for each reference nurse for training and feedback corresponded to the duration of the meetings; the time needed to collect information for each patient and to check and correct aberrant data was estimated with interviews. The time required for our team for training, support and analysis was estimated by using interviews. To estimate costs, only administrative and material costs were considered, not salaries.

## Results

Six nursing homes participated only in 2015 and were thus excluded from the analysis. Twelve participated in 2015 and 2016 and 15 participated from 2015 to 2017. The 12 nursing homes that participated for 2 years (NH2Y) had 994 patients in 2015 and 970 in 2016. The 15 nursing homes that participated for 3 years (NH3Y) had 1294, 1349 and 1357 patients in 2015, 2016 and 2017.

The mean age of NH2Y and NH3Y patients was 86.5 and 86.9 years; women represented 72.3% and 76.6%, respectively, and median length of stay was 2.1 and 2.2 years, respectively. Overall, 6.2% and 5.7% of NH2Y and NH3Y patients had a neuromotor impairment. Patients at greater risk of a PU had similar age, were more frequently female, stayed longer in the nursing home and had a neuromotor impairment than those with lower risk (Table 1).

**Table 1:** Patient characteristics and preventive measures by Braden risk score for pressure ulcer (PU) and duration of participation in the programme.

	Patients at low risk of PU			Patients at high risk of PU			p (2015–16)	p (2015–17)
	(Braden score ≥15)			(Braden score <15)				
	2015	2016	2017	2015	2016	2017		
**12 nursing homes participating in baseline and 1-year follow-up (NH2Y)**								
n	797	798		197	172			
Age (mean [SD])	86.2 (7.5)	87.0 (7.5)		86.1 (8.9)	85.7 (8.9)			
Sex = M (%)	233 (29.2)	220 (27.6)		48 (24.4)	42 (24.4)			
Length of stay (years—median [IQR])	1.7 [0.8, 3.7]	2.0 [1.0, 3.8]		3.1 [1.5, 4.7]	4.0 [2.4, 5.6]			
Multiple sclerosis (%)	1 (0.1)	2 (0.3)		2 (1.0)	2 (1.2)			
Hemiplegia (%)	23 (2.9)	20 (2.5)		30 (15.3)	29 (16.9)			
Paraplegia (%)	2 (0.3)	1 (0.1)		5 (2.6)	5 (2.9)			
Tetraplegia (%)	0 (0.0)	1 (0.1)		0 (0.0)	5 (2.9)			
Nutritional supplement (%)	41 (18.4)	36 (17.9)		37 (19.5)	41 (24.7)		0.63	
95% CI	13.5–24.1	12.9–23.9		14.2–26	18.3–32			
Patient repositioning (%)	/	/		54 (28.6)	97 (57.7)		**<0.001**	
95% CI				22.4–35.6	49.9–65.3			
Anti-decubitus bed or mattress (%)	167 (64.0)	171 (71.5)		96 (48.7)	116 (67.4)		**0.039**	
95% CI	57.8–69.8	65.4–67.2		41.8–56.2	59.9–74.4			
Dressing or moisturizing cream (%)	/	227 (95.0)		/	168 (97.7)			
95% CI		91.4–97.4			94.1–99.4			
**15 nursing homes participating in baseline, 1-year and 2-year follow-up (NH3Y)**								
n	1001	1077	1063	293	272	294		
Age (mean [SD])	86.8 (8.0)	86.7 (8.5)	87.3 (8.0)	86.7 (9.2)	87.0 (9.4)	86.9 (9.5)		
Sex = M (%)	252 (25.2)	250 (23.2)	263 (24.7)	59 (20.1)	54 (19.9)	57 (19.4)		
Length of stay (years—median [IQR])	1.9 [0.9, 3.9]	2.0 [0.8, 3.9]	2.0 [0.9, 3.9]	3.1 [1.4, 5.5]	3.1 [1.5, 5.6]	3.3 [1.5, 5.8]		
Multiple sclerosis (%)	2 (0.2)	5 (0.5)	2 (0.2)	4 (1.4)	2 (0.7)	3 (1.0)		
Hemiplegia (%)	26 (2.6)	30 (2.8)	30 (2.8)	24 (8.2)	24 (8.8)	33 (11.2)		
Paraplegia (%)	5 (0.5)	5 (0.5)	4 (0.4)	7 (2.4)	12 (4.4)	11 (3.7)		
Tetraplegia (%)	0 (0.0)	1 (0.1)	0 (0.0)	2 (0.7)	3 (1.1)	3 (1.0)		
Nutritional supplement (%)	44 (14.4)	76 (23.2)	64 (20.1)	82 (29.5)	82 (32.3)	85 (31.6)	0.069	0.23
95%CI	10.7–18.9	18.8–28.2	15.8–24.9	24.2–35.2	26.6–38.4	21.6–37.5		
Patient repositioning (%)	/	/	/	96 (33.6)	116 (43.6)	122 (41.9)	**0.003**	**0.031**
95% CI				28.1–39.4	37.6–49.8	36.2–47.8		
Anti-decubitus bed or mattress (%)	201 (54.8)	224 (57.6)	273 (72.2)	178 (60.8)	182 (66.9)	214 (72.8)	0.31	**0.017**
95% CI	49.4–56.8	52.5–62.5	67.4–76.7	54.9–66.4	61–72.5	67.3–77.8		
Dressing or moisturizing cream (%)	/	354 (91.0)	353 (93.4)	/	261 (96.0)	277 (94.2)		
95% CI		87.7–93.6	90.4–95.7		92.9–98	90.9–96.6		

IQR, interquartile range; 95% CI, 95% confidence interval.

### Preventive measures

From 2015 to 2016, the NH2Y group showed significant improvement in “patient repositioning” (from 28.6% to 57.7%, p <0.001) and “anti-decubitus bed or mattress” (from 57.4% to 69.8%, p 0.039) as well as “nutritional supplement” but not significantly (from 18.9% to 21%, p 0.63) (Table 1, top panel). The NH3Y group showed analogous improvements from 2015 to 2017 (“patient repositioning” from 33.6% to 41.9%, p 0.031; “anti-decubitus bed or mattress” from 57.4% to 72.4%, p 0.017; “nutritional supplement” from 21.6% to 25.3%, p 0.23). Dressings or moisturizing creams were used for almost all patients at risk in the two groups (Table 1, bottom panel).

### Variability in preventive measures

The amplitude of the IQR (a measure of variability which corresponds to the difference between the third and first quartiles) of preventive measures in NH2Y and NH3Y is reported in Table 2. The NH2Y group showed reduced variability in all preventive measures, whereas the NH3Y group showed reduced nutritional supplement and anti-decubitus bed or mattress variability.

**Table 2:** Interquartile ranges (Q3–Q1) (%) for preventive measures by duration of participation in the programme.

	2015	2016	2017
**12 nursing homes participating in baseline and 1-year follow-up (NH2Y)**			
Nutritional supplement	19 (31–12)	12 (25–13)	
Patient repositioning	57 (63–6)	34 (74–40)	
Anti-decubitus bed or mattress	52 (88–36)	45 (95–50))	
**15 nursing homes participating in baseline, 1-year and 2-year follow-up (NH3Y)**			
Nutritional supplement	25 (36–11)	28 (42–14)	9 (29–20)
Patient repositioning	39 (60–21)	42 (74–32)	40 (69–29)
Anti-decubitus bed or mattress	53 (88–35)	53 (93–40)	38 (94–56)

### Pressure ulcers

The NH2Y group showed a non-significant reduction in prevalence of acquired PUs (from 3.9% to 2.6%, p 0.24) and acquired category 2–4 PUs (from 2.2% to 1.5%, p 0.38; Table 3, top panel). After 1 year, the NH3Y group showed a non-significant reduction in acquired PUs (from 4.5% to 3.7%, p 0.54) and acquired category 2–4 PUs (from 2.7% to 2.4%, p 0.87). After 2 years, the reduction was significant for acquired PUs (from 4.5% to 2.9%, p 0.035) and for category 2–4 acquired PUs (from 2.7% to 1.5%, p 0.043; Table 3, bottom panel). Nursing home size and length of stay did not affect NH2Y and NH3Y outcomes.

**Table 3:** PU prevalence by Braden risk score and duration of participation in the programme.

	Patients at low risk of PU (Braden score ≥15)			Patients at high risk of PU (Braden score <15)			p (2015–16)	p (2015–17)
	2015	2016	2017	2015	2016	2017		
**12 nursing homes participating in baseline and 1-year follow-up (NH2Y)**								
Acquired PUs (%)	17 (2.1)	10 (1.3)		23 (11.7)	15 (8.7)		0.239	
95% CI	1.2–3.4	0.6–2.3		7.2–16.5	5–14			
Acquired PUs (categories 2–4) (%)	9 (1.1)	6 (0.8)		13 (6.6)	9 (5.2)		0.376	
95% CI	0.5–2.1	0.3–1.6		3.6–11.1	2.4–9.7			
**15 nursing homes participating in baseline, 1-year and 2-year follow-up (NH3Y)**								
Acquired PUs (%)	19 (1.9)	17 (1.6)	16 (1.5)	39 (13.3)	33 (12.1)	23 (7.8)	0.54	**0.035**
95% CI	1.1–2.9	0.9–2.5	0.9–2.4	9.6–17.7	8.5–16.6	5–11.5		
Acquired PUs (categories 2–4) (%)	11 (1.1)	11 (1.0)	7 (0.7)	24 (8.2)	22 (8.1)	13 (4.4)	0.87	**0.043**
95% CI	0.5–2	0.5–1.8	0.3–1.3	5.3–12	5.1–12	2.4–7.4		

**Fig 1:**
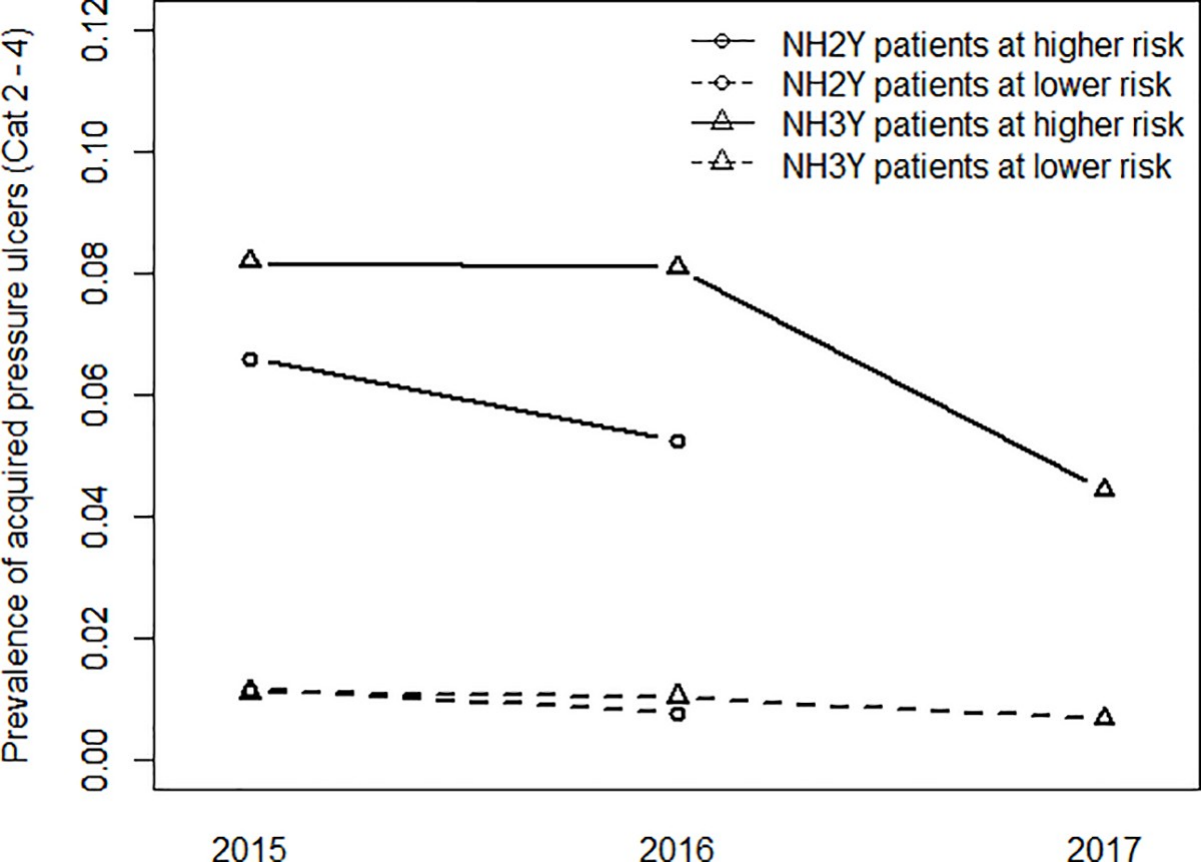
Evolution of acquired pressure ulcer (PU) prevalence (category 2–4) by Braden risk score and duration of participation in the programme.

Fig 1 illustrates the trends of NH2Y and NH3Y according to Braden risk level. The NH2Y group showed reduced percentage (prevalence difference) of acquired PUs (categories 2–4) for patients at low risk, by 0.3%, and for patients at high risk, by 1.4%. The NH3Y group showed a reduction by 0.4% and 3.8% for patients at low and high risk, respectively.

**Fig 2:**
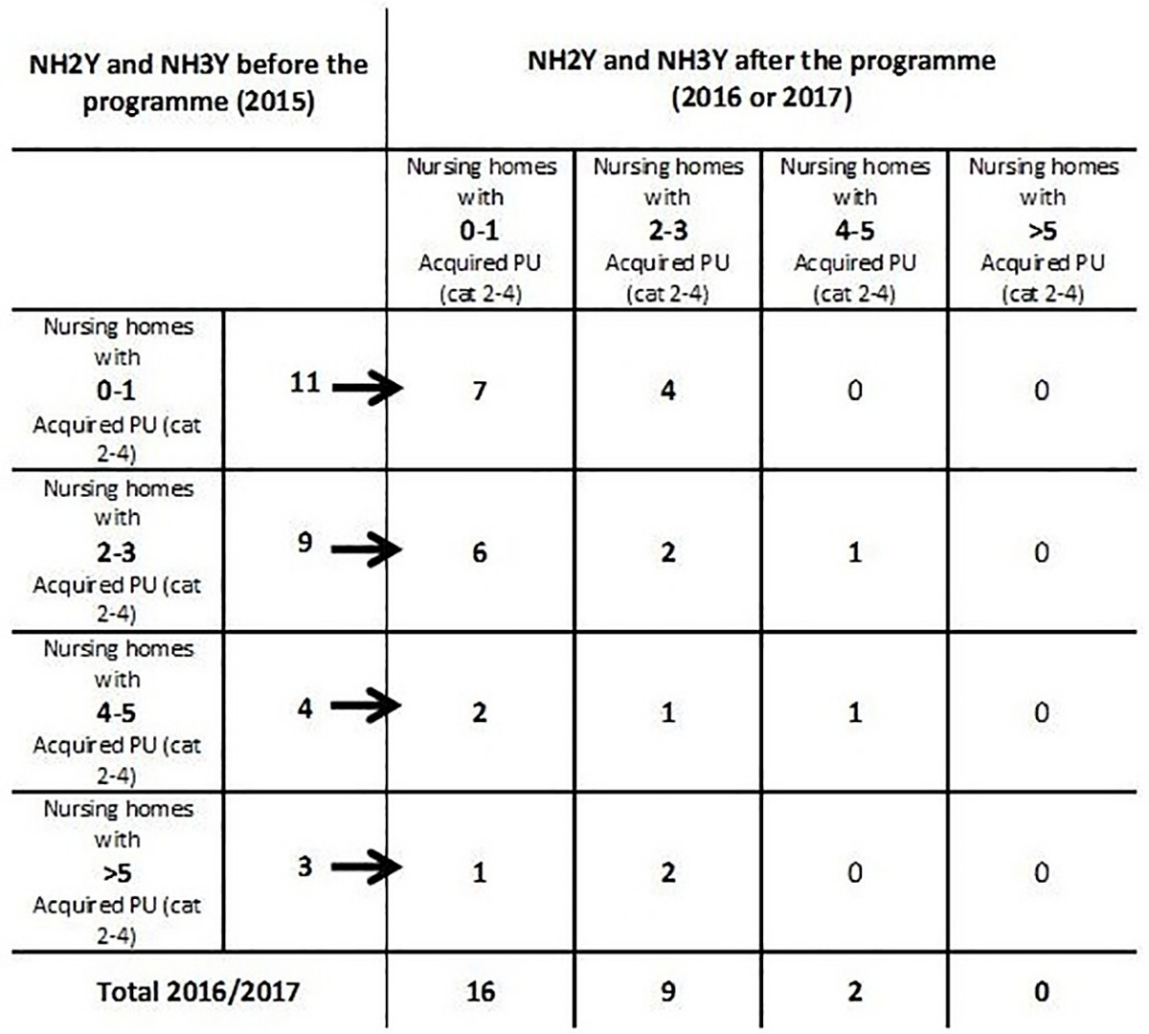
Evolution of the number of patients with acquired PU prevalence (category 2–4) per nursing home.

PU prevalence decreased especially among the nursing homes with the most patients with PUs. In 2015, the four less-performing nursing homes in the NH3Y group had 25 category 2–4 acquired PUs, whereas in 2017, just one nursing home had > 2 category 2–4 acquired PUs. In the NH2Y group, three nursing homes had > 2 category 2–4 acquired PUs in 2015 and one in 2016. Fig 2 shows the number of category 2–4 acquired PUs per nursing home before and after the programme in NH2Y and NH3Y groups.

### Pressure ulcer variability

The amplitude of IQR for acquired PU prevalence per nursing home decreased from 2.1% (range 4.5% to 2.4%) in 2015 to 1.5% (range 3% to 1.5%) in 2016 in the NH2Y group and from 4.7% (range 5.8% to 1.1%) in 2015 to 3.6% (range 5.4% to 1.8%) in 2016 and 1.3% (range 3% to 1.7%) in 2017 in the NH3Y group.

### Costs

Concerning implementation, for every reference nurse, audit and feedback took 2 hr on average for training; 10 min per patient for gathering information before and during the survey; 1 hr to check and correct aberrant data and 2 hr for feedback. Every year, our team spent half a day on average to prepare the course, 2 hr to train staff, 1 day to oversee the survey, 1 day to clean data, 2 days for statistical analysis and writing reports, and half a day to prepare and give feedback.

Our team asked for a compensation of approximately 5 € per patient for the initial setup of the survey and analysis and in the next years, asked 2 € per patient.

## Discussion

This 3-year prospective longitudinal study of nursing home residents from 27 institutions participating for 2 (NH2Y) or 3 (NH3Y) years, shows the implementation of an audit and feedback regional programme to enhance adherence to preventive practices and reduce acquired PU prevalence in nursing homes.

### Preventive practices

Good results were achieved at 1- and 2-year follow-up, by significantly increasing “patient repositioning” (from 28.6% to 57.7% and from 33.6% to 41.9%) and “anti-decubitus bed or mattress” (from 57.4% to 69.8% and from 57.4% to 72.4%). These are widely recommended, beneficial [19–21] and efficient [21,22] preventive measures.

Previous studies in Europe found relatively variable results in preventive measures, with nutrition intervention ranging from 21.7% of nursing home patients in The Netherlands to 71.3% in Germany, and repositioning at the required frequency ranging from 9% in Ireland to 19.6% in The Netherlands and 47% in Germany. The use of pressure-relieving mattresses ranged from 43.3% to 63.6% in the mentioned countries [23,24]; a suboptimal use of pressure-relieving mattresses was also reported in a Norwegian study [25]. The data for “nutritional supplement” in our NH2Y and NH3Y groups agreed with Dutch values and were significantly lower than that for German nursing homes. “Repositioning” and “anti-decubitus bed or mattress” data in 2015 in NH2Y and NH3Y groups were better than in Ireland and The Netherlands but not as good as in Germany. In the last year of participation, the percentages agreed with or were better than that for German nursing homes.

The programme also had a strong impact on variability. Patient repositioning variability was lowered the most in NH2Y groups, whereas NH3Y groups showed a marked decrease in the use of anti-decubitus mattresses and nutritional supplement variability.

Even if we consider these results satisfactory, variation and underuse of good clinical practices were still high at the end of the programme.

### Pressure ulcers

In the literature, acquired PU is often considered the preferred outcome to assess PU reduction [17,26–30]. In 2015, the prevalence of category 2–4 acquired PU was 2.2% in NH2Y groups and 2.7% in NH3Y groups. In the last year of participation, it was 1.5% in both groups.

In Europe, nursing-home PU prevalence was 31.4% in The Netherlands [31], 14.5% in Sweden [32], 9% in Ireland [24] and 6.4% in Germany [31]. In the United States, category 2–4 PU prevalence in nursing homes ranged from 11% in the west north central region to 21% in the middle atlantic region [33]. At baseline, the NH2Y and NH3Y overall PU prevalence (categories 1–4) was 5.6% and 5.4%, respectively (data not reported in results). Even before the programme, PU prevalence in Geneva nursing homes was lower than in the United States, Germany, Sweden or The Netherlands and with the programme, this difference became more pronounced. Similar to preventive measures, the programme helped reduce acquired PU prevalence variability: IQR values decreased from 2.5% to 2.1% in NH2Y groups and from 5.4% to 1.9% in NH3Y groups.

At 1-year follow-up, both groups showed enhanced adherence to preventive practices and showed reduced, albeit not significantly, facility-acquired PU, however, better results were achieved for the NH2Y than NH3Y group, for which PU prevalence remained stable. A possible explanation for this difference is selection bias. Nursing homes considering that the programme had already helped them improve may have decided to quit the programme, seeing no additional value in staying longer.

Nursing home size and length of stay did not affect the programme’s outcomes. This result differs from previous studies often considering that the number of beds is linked to PU prevalence [9,10,34]. This finding may be due to the relatively small size of included nursing homes (maximum size 230 beds). It suggests no structural barriers to improvement: regardless of the starting point, each institution can improve.

Several reasons may explain the success of the programme. A Cochrane multivariable meta-regression indicated five elements for success of a programme: 1) feedback may be more effective when baseline performance is low, 2) the person responsible is a supervisor or colleague, 3) the progrmme is delivered in both verbal and written formats, 4) the programme is provided more than once, and 5) the programme includes both explicit targets and an action plan [16]. In our study, we found four of the five elements. 1) The 2- and especially 3-year programme was particularly effective for nursing homes with the highest number of acquired PUs and for patients at high risk. 2) Our feedback was given by a well-reputed nurse expert in quality improvement, and every reference nurse was experienced, capable of transmitting feedback to colleagues in the nursing home. Thus, the people responsible were both a supervisor from an external institution and respected colleagues. 3) During the feedback session, two written reports were given and results were also relayed orally. 4) Some days after the feedback session, the head of the Geneva nursing home association sent the written reports by e-mail. Including targets and action plans would have been difficult because we did not have a specific authority to do so. Indeed, the head of the Geneva nursing home association asked the nurse in charge of measuring PUs in the university hospitals to help them measure prevalence, thereby allowing the nursing homes the freedom to establish their own targets and plan their improvement actions. Furthermore, participation was voluntary. However, our realistic hope was that every reference nurse set the lowest PU prevalence as the target. During the 2017 feedback session, we asked participants about, in their opinion, the strengths of the program. The most common answers were push for training, criteria for prevention targets, criteria for equipment purchase, motivation and refocus on problematic situations. Respondents of this oral survey were from the NH3Y group only.

### Costs

Although the programme seems promising, its costs must be determined. Every year, the audit and feedback process takes about 18 hr of work for a medium-sized nursing home (80 beds) and 5 days for 1 or 2 members of our team.

Although the time for examining patients, training staff, and providing feedback cannot be reduced, the time needed to check for aberrant data and produce reports can be greatly decreased by using automatic software. For the survey with aberrant data checks, we now use Redcap, a free electronic data capture software, and this survey is available upon request. For the automatic reports, we use R, an open-source statistical software, and the code is available upon request. Concerning implementation financial costs, each nursing home was asked for 5 € per patient for the initial setup of the survey and analysis, and 2 € per patient for the next years. Thus, the price would not be a barrier for participating in the programme because it represents only 400 (year 1) + 160 (year 2) + 160 (year 3) € for a medium-sized nursing home.

## Limitations

The main limitation is that we used a point-prevalence measure to test our programme efficacy: PU severity may have changed between the time it appeared and the time it was evaluated. A second limitation is not having tested inter-rater reliability of reference nurses. Discrepancies in PU staging are possible, but most discrepancies in assessment correspond to a difference of one category [35]. Analyzing all PUs together (or excluding category 1) reduced the risk of this bias. Misclassification bias was also possible: reference nurses could underreport PU to make their nursing home or their managers look better. The fact that our study used anonymous feedback, voluntary participation and no financial penalties should have mitigated this risk.

## Conclusions

Variation and underuse of good clinical practices are a problem in PU prevention and management in nursing homes. Audit and feedback is relatively easy to implement at the regional level and could enhance adherence to preventive measures and reduce PU prevalence in nursing homes. The effectiveness is greater for patients at increased risk and for nursing homes with increased number of PUs at baseline. We advise not interrupting the programme after 1 year, especially if the results are not optimal.

## Supporting information

S1 Data (XLSX)
